# Exposure–Response Analysis of Cardiovascular Outcome Trials With Incretin-Based Therapies

**DOI:** 10.3389/fendo.2022.893971

**Published:** 2022-05-26

**Authors:** Qi Pan, Mingxia Yuan, Lixin Guo

**Affiliations:** ^1^ Department of Endocrinology, Beijing Hospital, National Center of Gerontology, Institute of Geriatric Medicine, Chinese Academy of Medical Science, Beijing, China; ^2^ Department of Endocrinology, Beijing Friendship Hospital, Capital Medical University, Beijing, China

**Keywords:** type 2 diabetes mellitus, cardiovascular outcome trials, glucagon-like peptide-1 receptor agonist, dipeptidyl peptidase-4 inhibitor, incretin

## Abstract

Our study aimed to evaluate the exposure–response relationship between incretin-based medications and the risk of major adverse cardiovascular events (MACE) using cardiovascular outcome trials (CVOTs). Eleven CVOTs with incretin-based medications were included. The median follow-up time, percentage of time exposure, and hazard ratio (HR) of MACE were obtained from each CVOT. The pharmacokinetic parameters of glucagon-like peptide-1 receptor agonists (GLP-1 RAs) and dipeptidyl peptidase-4 inhibitor (DPP-4) were obtained from published studies. Regression analysis was performed to assess the relationship between drug exposure and MACE HR. Cutoff values were determined from the ROC curves. The linear regression results indicated that log C_max_, log AUC_0–24h_, and log AUC_CVOT_ are negatively correlated with MACE HR (R^2^ = 0.8494, R^2^ = 0.8728, and R^2^ = 0.8372, respectively; all p < 0.0001). The relationship between drug exposure (log C_max_, log AUC_0–24h,_ and log AUC_CVOT_) and MACE HR strongly corresponded with the log (inhibitor) *vs*. response curve (R^2^ = 0.8383, R^2^ = 0.8430, and R^2^ = 0.8229, respectively). The cutoff values in the ROC curves for log C_max_, log AUC_0–24h_, and log AUC_CVOT_, were 2.556, 3.868, and 6.947, respectively (all p = 0.007). A Fisher’s exact test revealed that these cutoff values were significantly related to cardiovascular benefits (all p < 0.05). Our study revealed a linear exposure–response relationship between drug exposure and MACE HR. We conclude that the cardiovascular benefits of incretin-based therapies may occur with higher doses of GLP-1 RAs and with increased exposure.

## Introduction

Type 2 diabetes (T2DM) is frequently accompanied by various cardiovascular complications. Further, cardiovascular complications are the leading cause of disability and death in patients with T2DM (1). Numerous *in vitro* studies and animal studies have investigated the effects of dipeptidyl peptidase-4 (DPP-4) inhibitors and GLP-1 receptor agonists (GLP-1 RAs) on the cardiovascular system (2, 3). Considering the results of cardiovascular outcome trials (CVOTs) with incretin-based medications, significant heterogeneity in cardiovascular effects could be found between DPP-4 inhibitors and GLP-1 RAs. While the results obtained with DPP-4 inhibitors (alogliptin, saxagliptin, sitagliptin, and linagliptin) demonstrated cardiovascular safety (4–7), the majority of GLP-1 RAs (including liraglutide, albiglutide, dulaglutide, and subcutaneous semaglutide) significantly decreased the risk of major adverse cardiovascular events (MACE) (8–11). Furthermore, the meta-analysis results suggested that the GLP-1RA class could reduce the risk of MACE, cardiovascular mortality, and all-cause mortality (12). However, the results of the ELIXA study with lixisenatide (cardiovascular safety) (13), the PIONEER 6 study with oral semaglutide (cardiovascular safety) (14), and the EXSCEL study with extended exenatide (a trend toward reduction in MACE) (15), cast doubt on the existence of a class effect for GLP-1 RAs.

Although our overall understanding of the cardiovascular benefits of GLP-1RAs has evolved over time, head-to-head comparisons between different incretin-based drugs in the completed CVOTs have been lacking. Hence, differences in baseline characteristics/trial execution may account for the divergent MACE results. However, evidence from several studies suggest associations between cardiovascular risk reduction and HbA1c reduction (16), non-glycemic effects (17), or time of exposure to GLP-1 RAs (18). In the present study, we evaluated the exposure–response relationship of incretin-based medications by determining the effects of drug exposure on MACE risk in cardiovascular outcome trials.

## Research Design and Methods

In total, 11 CVOTs comparing add-on therapy using a DPP-4 inhibitor or GLP-1RA with a placebo were included. HbA1c reduction, weight loss, median follow-up time, percentage of time exposure to the trial drug, and hazard ratio (HR) of 3-point MACE in each individual CVOT were obtained from the records. Although the plasma concentration of GLP-1 RAs or GLP-1 was not measured in the CVOTs, the clinical pharmacokinetic and pharmacodynamic properties of these trial drugs have previously been established and published. The maximum observed plasma concentration of GLP-1 RA or active GLP-1 (C_max_), the mean area under the curve (AUC_0–24h_) with multiple doses, and the total AUC in CVOT (AUC_CVOT_) were used to assess drug exposure (**Table 1**). AUC_CVOT_ was calculated by substituting the median follow-up time and percentage of time exposure to the trial drug using Eq. 1, as follows:


(1) $${\text{AUC}}_{\text{CVOT}}={\text{AUC}}_{0-24\text{h}}\times \text{Median follow}-\text{up time }\times \text{Percentage of time exposure to trial drug }$$

**Table 1:** Drug exposure related parameters.

Parameters	Description
C_max_	The maximum observed plasma concentration of GLP-1 RA or active GLP-1 with multiple doses. C_max_ represents exposure concentration of drug.
AUC_0–24h_	The mean drug exposure is expressed as the mean area under the curve from 0–24 hours. AUC_0–24h_ represents mean exposure concentration and time of drug.
AUC_CVOT_	Total drug exposure is expressed as the area under the curve during the median follow-up period of the individual CVOT. AUC_CVOT_ represents total exposure concentration and time of drug.

Measurements of normalized cyclic adenosine monophosphate (cAMP) production, induced by GLP-1 RAs (19), was used to calibrate C_max_, AUC_0–24h_, and AUC_CVOT_. Using the calibrated C_max_, calibrated AUC_0–24h_, and calibrated AUC_CVOT_, a sensitivity analysis was conducted to assess the exposure–response relationship.

## Statistical Analyses

The values of C_max_, AUC_0–24h_, and AUC_CVOT_ were obtained as continuous variables and analyzed after natural logarithmic transformation. Linear regression analysis was used to assess the relationship between drug exposure (log C_max_, log AUC_0–24h_, or log AUC_CVOT_) and MACE HR, HbA1c reduction, and weight loss. Nonlinear regression analysis was used to examine the exposure–response relationship by fitting a log (inhibitor) *vs*. response curve. Continuous variables were evaluated using receiver operating characteristic (ROC) curves. Cutoff values were determined following an assessment of the ROC curves. Fisher’s exact tests were used to compare categorical variables. A p value < 0.05 was used to determine statistical significance. All analyses were performed using GraphPad Prism 6 (GraphPad Software, San Diego, CA, USA).

## Results

### Drug Exposure

C_max_, AUC_0–24h_, and AUC_CVOT_ of GLP-1 RAs and native GLP-1 are reported in **Table 2**. According to the pharmacodynamic studies of alogliptin, saxagliptin, sitagliptin, and linagliptin, the DPP4 inhibitors significantly increased native GLP-1 levels in patients with T2DM (compared with placebo), yielding an average level of ≤ 19.0 pmol/L (**Table 2** and **Figure S1**) (20–23).

**Table 2:** Exposure–response related parameters.

	MACE HR	Mean follow up (years)	Percentage of time exposure to trial drug	C_max_ (pmol/L)	AUC_0–24h_ (pmol·h/L)	AUC_CVOT_ (pmol·h/L)
EXAMINE (Alogliptin) (4, 20)	0.96	1.5	0.97	14.2^ac^	132	70155
SAVOR-TIMI (Saxagliptin) (5, 21)	1.00	2.1	1.00	4.8 ^acd^	72^d^	55188†
TECOS (Sitagliptin) (6, 22)	0.98	3.0	1.00	19.0 ^ac^	191	208926
CARMELINA (Linagliptin) (7, 23)	1.02	2.2	0.86	11.8 ^ac^	136	93643
ELIXA (Lixisenatide) (13, 24)	1.02	2.1	0.88	38.5 ^ae^	175	117704
EXSCEL (Exenatide OW) (15, 25)	0.91	3.2	0.76	71.4^be^	1717	1523791
LEADER (Liraglutide) (8, 26)	0.87	3.8	0.84	22000.0^ae^	524000	610501920
HARMONY (Albiglutide) (9, 27)	0.78	1.6	0.87	29178.0 ^be^	622309	316182858
SUSTAIN-6 (Semaglutide 1.0 mg) (11, 28)	0.71	2.1	0.87	30000.0^be^	719143	479564039
SUSTAIN-6 (Semaglutide 0.5 mg) (11, 28)	0.77	2.1	0.87	15800.0^be^	380429	253690714
REWIND (Dulaglutide) (10, 29)	0.88	5.4	0.82	1810.0^be^	31746	51308520
PIONEER 6 (Oral semaglutide) (14, 30)	0.79	1.3	1.00	14600.0^ac^	283700	137722165

a once daily.

b once weekly.

c oral administration.

d the raw data were obtained from the visual graph of the published paper.

e subcutaneous injection.

Pharmacokinetic studies of GLP-1 RAs revealed that the plasma concentrations of GLP-1 RAs in T2DM patients varied significantly. The peak plasma concentration of lixisenatide was 187.2 pg/mL (38.5 pmol/L) after multiple daily injections (24), while the steady-state plasma concentration of once-weekly exenatide reached 300 pg/mL (71.4 pmol/L) after multiple dosing (25). In contrast, several different pharmacokinetic studies have revealed that the steady-state concentrations of other GLP-1 RAs (including dulaglutide, liraglutide, albiglutide, subcutaneous semaglutide, and oral semaglutide) reach substantially higher (nanomolar) levels (**Table 2** and **Figure S1**) (26–30).

### MACE Risk in CVOTs

According to the results of our statistical analysis of the primary outcomes in the CVOTs, several of the add-on therapies demonstrated cardiovascular noninferiority when compared with placebo. These included alogliptin [EXAMINE trial, HR, 0.96; p < 0.001 for noninferiority and p = 0.32 for superiority] (4), saxagliptin (SAVOR-TIMI 53 trial, HR, 1.00; p < 0.001 for noninferiority and p = 0.99 for superiority) (5), sitagliptin (TECOS trial, HR, 0.98; p < 0.001 for noninferiority and p = 0.65 for superiority) (6), linagliptin (CARMELINA trial, HR, 1.02; p < 0.001 for noninferiority and p = 0.74 for superiority) (7), lixisenatide (ELIXA trial, HR, 1.02; p < 0.001 for noninferiority and p = 0.81 for superiority) (13), once-weekly exenatide (EXSCEL trial, HR, 0.91; p < 0.001 for noninferiority and p = 0.06 for superiority) (15), and oral semaglutide (PIONEER 6 trial, HR, 0.79; p < 0.001 for noninferiority and p = 0.17 for superiority) (14).

Conversely, liraglutide (LEADER trial, HR, 0.87; p < 0.001 for noninferiority and p = 0.01 for superiority) (8), albiglutide (HARMONY trial, HR, 0.78; p < 0.0001 for noninferiority and p = 0.0006 for superiority) (9), dulaglutide (REWIND trial, HR, 0.88; p = 0.026 for superiority) (10), and subcutaneous semaglutide (SUSTAIN-6 trial, HR, 0.74; p < 0.0001 for noninferiority and p = 0.02 for superiority) (11) all demonstrated cardiovascular superiority over placebo (**Table 2**).

### Exposure–Response Relationship

**Figure 1:**
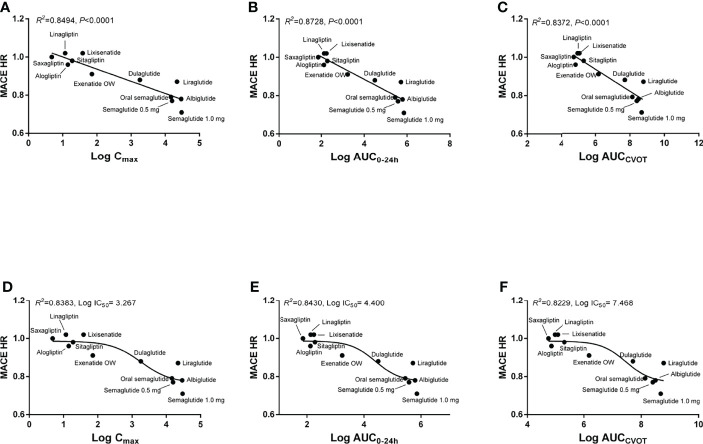
Correlation between drug exposure and MACE HR. **(A)** Linear regression analysis between log C_max_ and MACE HR; **(B)** Linear regression analysis between log AUC_0–24h_ and MACE HR; **(C)** Linear regression analysis between log AUC_CVOT_ and MACE HR; **(D)** Nonlinear regression analysis between log C_max_ and MACE HR; **(E)** Nonlinear regression analysis between log AUC_0–24h_ and MACE HR; **(F)** Nonlinear regression analysis between log AUC_CVOT_ and MACE HR.

**Figure 2:**
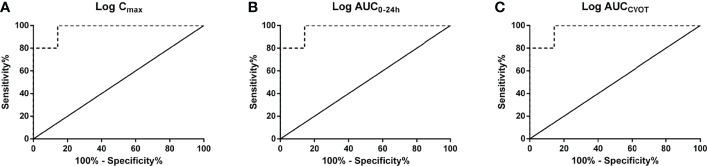
Receiver operating characteristic (ROC) curves. **(A)** log C_max_; **(B)** log AUC_0–24h_; **(C)** log AUC_CVOT_.

The linear regression results demonstrate that log C_max_, log AUC_0–24h_, and log AUC_CVOT_ negatively correlate with MACE HR (R^2^ = 0.8494, R^2^ = 0.8728, and R^2^ = 0.8372, respectively; p < 0.0001; **Figures 1A–C**). The relationship between drug exposure (log C_max_, log AUC_0–24h_, or log AUC_CVOT_) and MACE HR showed a good correspondence with the fitted curve (R^2^ = 0.8383; R^2^ = 0.8430; R^2^ = 0.8229, respectively; **Figures 1D–F**). The ROC curve was used to evaluate drug exposure and define cutoff values (**Figure 2**). For the log C_max_, log AUC_0–24h_, and Log AUC_CVOT_ ROC curves, the cutoff values were 2.556, 3.868, and 6.947, respectively (all p = 0.007). Detailed results reporting areas under the curve, sensitivity, and specificity are shown in **Table 3**. These cutoff values were all significantly related to cardiovascular superiority (all p < 0.05, Fisher’s exact test).

**Table 3:** ROC Curves of all continuous variables.

Variable	Cutoff	P Value	Areas under the curve	Sensitivity	Specificity
Log C_max_	2.556	0.007	0.971	100.0	85.7
Log AUC_0–24h_	3.868	0.007	0.971	100.0	85.7
Log AUC_CVOT_	6.947	0.007	0.971	100.0	85.7

A similar correlation existed in the secondary prevention cohorts with a history of CVD (p < 0.01; **Figure S2**), but not in the primary prevention cohorts without a history of CVD (**Supplementary Figure S3**). The relationships between drug exposure and HbA1c reduction (compared with placebo) and between drug exposure and weight loss (compared with placebo) were also examined (**Supplementary Figures S4, S5**). In both instances, statistically significant relationships were observed (p < 0.01). A sensitivity analysis was also performed on calibrated C_max_, calibrated AUC_0–24h_, and calibrated AUC_CVOT_. The results were generally consistent with the results of the aforementioned analysis (**Supplementary Figures S6–S10** and **Table S1**).

## Discussion

In healthy subjects, basal plasma levels of native GLP-1 are generally below 10 pmol/L, while postprandial levels of GLP-1 rise to 10–30 pmol/L (31–33). In comparison, prediabetes or T2DM subjects generally demonstrate lower basal GLP-1 levels, and/or a reduction in GLP-1 response to oral glucose load (33). DPP4 inhibitors reportedly raise the postprandial levels of native GLP-1 approximately 2–4 fold (20, 34). However, the concentrations of most GLP-1 RAs (except lixisenatide and once-weekly exenatide) are substantially higher than the reported physiological concentrations of GLP-1.

Our results indicate that the exposure concentrations of DPP4 inhibitor (lixisenatide) and once-weekly exenatide associated with cardiovascular safety are low, as the concentrations of these drugs were close to physiological levels (picomolar levels). In contrast, the exposure concentrations of other GLP-1 RAs (except oral semaglutide), which are associated with cardiovascular benefits, are higher (nanomolar levels). A strong relationship between the exposure concentration and MACE HR was demonstrated through regression analysis (p < 0.0001). Moreover, a good fit was obtained between the observed relationship and the theoretically constructed model. The exposure–response relationship of GLP-1 was also demonstrated in a cross-sectional study, and linear regression analysis showed that higher glucose-stimulated GLP-1 levels were associated with clinically relevant lower blood pressure (associated with beneficial effects on the cardiovascular system) (35). In addition, a strong relationship was demonstrated between the mean and total drug exposure and MACE HR.

Drug exposure is dependent on both concentration and time. When only concentration was considered, our ROC results suggest that log C_max_ > 2.556 (i.e., C_max_ > 359.7 pmol/L) could be a predictor for cardiovascular benefits. However, when both concentration and time were considered, our results indicate that log AUC_0–24h_ > 3.868 and log AUC_CVOT_ > 6.947 could be predictors for cardiovascular superiority. These results suggest that the drug should be continuously used at a steady-state C_max_ of 307.5 pmol/L per day for 3.29 years consecutively (the value was obtained by dividing AUC_CVOT_ by AUC_0–24h_). Native GLP-1 levels when using DPP4 inhibitors and GLP-1 RA levels (lixisenatide and once-weekly exenatide) were both less than the predicted C_max_ values. Hence, these regimens show cardiovascular safety only. For PIONEER 6, the median exposure time was only 15.9 months. We predict that better results may be obtained by extending the exposure time with oral semaglutide.

The extent to which exendin-4-based agonists differ in cardiovascular effects from GLP-1-based agonists has been extensively debated. Although CVOTs do not provide evidence of any cardiovascular benefits of using exendin-4-based agonists, there is no evidence to suggest that exendin-4-based agonists attenuate activation of GLP-1 receptor signaling (2). The present study demonstrates significant associations between GLP-1 RA drug exposure and HbA1c reduction and between GLP-1 RA drug exposure and weight loss. HbA1c reduction and weight loss may both play important roles in mediating MACE benefits (16, 36). Compared with other GLP-1 RAs, the therapeutic benefits (HbA1c reduction, weight loss, and cardiovascular benefits) of using recommended and approved doses of lixisenatide or once-weekly exenatide, which reflect a tradeoff between the adverse effects and the therapeutic benefits (HbA1c reduction and weight loss) observed in phase 2–3 studies, are significantly reduced (37). Suboptimal GLP-1 RA drug exposure with exendin-4-based agonists may be a critical cause of their lack of cardiovascular benefits (similar time exposure, but lower dose exposure).

The importance of supraphysiological doses of GLP-1 was originally proposed by J.J. Holst (38). Holst considered moderately elevated GLP-1 concentrations to have a significant effect on pancreatic islets, higher concentrations to slow gastric emptying and reduce food intake, and much higher concentrations to lead to side effects (nausea, diarrhea, and vomiting) (38). Our study provides evidence to support the notion that higher GLP-1 RA drug exposure is associated with additional cardiovascular benefit.

This study has several limitations. The parameters for drug exposure were obtained from different studies and may be biased. Although the parameters for drug exposure were calibrated using normalized cAMP, a degree of bias may be inevitable. In addition, our study is based on trial level analyses using the published literature, and not on patient level analyses. Thus, some inferences based on these results may ultimately prove to be misleading.

In conclusion, our study demonstrates a good exposure–response relationship between drug exposure and MACE HR. Our results suggest that the cardiovascular benefits of incretin-based therapies may occur with higher exposure to GLP-1 RAs.

## Data Availability Statement

The original contributions presented in the study are included in the article/**Supplementary Material**. Further inquiries can be directed to the corresponding author.

## Author Contributions

QP, MY, contributed to data collection and analysis. LG contributed to the study design and interpretation. All authors approved the manuscript. All authors contributed to the article and approved the submitted version.

## Funding

This work was supported by the China International Medical Foundation (grant Z-2017-26-1902).

## Conflict of Interest

The authors declare that the research was conducted in the absence of any commercial or financial relationships that could be construed as a potential conflict of interest.

## Publisher’s Note

All claims expressed in this article are solely those of the authors and do not necessarily represent those of their affiliated organizations, or those of the publisher, the editors and the reviewers. Any product that may be evaluated in this article, or claim that may be made by its manufacturer, is not guaranteed or endorsed by the publisher.
