# Supplementary material for: Exposure–Response Analysis of Cardiovascular Outcome Trials With Incretin-Based Therapies
Source: Front Endocrinol (Lausanne). 2022 May 26;13:893971. doi: 10.3389/fendo.2022.893971 (PMC9204533; doi:10.3389/fendo.2022.893971)
Supplement: Supplementary file 1 [file DataSheet_1.docx]

**Supplementary Information**

**Exposure-response analysis of cardiovascular outcomes trials with incretin-based therapies**

**Running title**: Exposure-response relationship in CVOTs

Qi Pan^1#^, Mingxia Yuan^2#^, Lixin Guo^1^

^1^ Department of Endocrinology, Beijing Hospital, National Center of Gerontology, Institute of Geriatric Medicine, Chinese Academy of Medical Science, Beijing, China.

^2^ Department of Endocrinology, Beijing Friendship Hospital, Capital Medical University, Beijing, China.

***Correspondence:**

Lixin Guo, MD, PhD

E-mail: glx1218@163.com


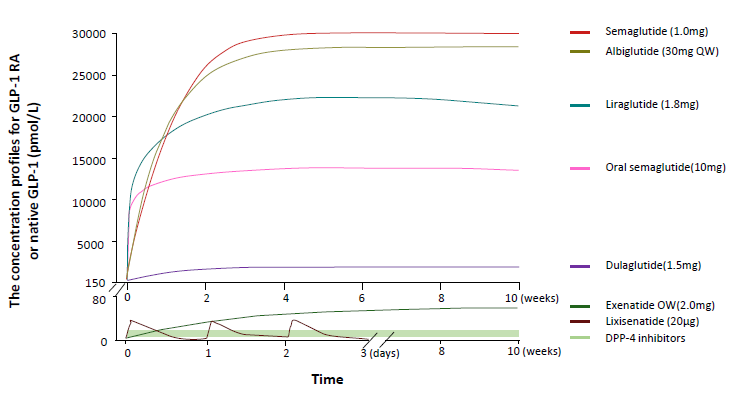


**Figure S1. The concentration profiles for GLP-1 RA and native GLP-1 with multiple doses at steady-state.** The concentrations of native GLP-1 after DPP4 inhibitor treatment were expressed through an approximate range (4.8–19.0 pmol/L).

**

**

**Figure S2. Correlation between drug exposure and MACE HR with CVD.** (A) Linear regression analysis between log C_max_ and MACE HR with CVD; (B) Linear regression analysis between Log AUC_0–24h_ and MACE HR with CVD; (C) Linear regression analysis between log AUC_CVOT_ and MACE HR with CVD; (D) Nonlinear regression analysis between log C_max_ and MACE HR with CVD; (E) Nonlinear regression analysis between log AUC_0–24h_ and MACE HR with CVD; (F) Nonlinear regression analysis between log AUC_CVOT_ and MACE HR with CVD.


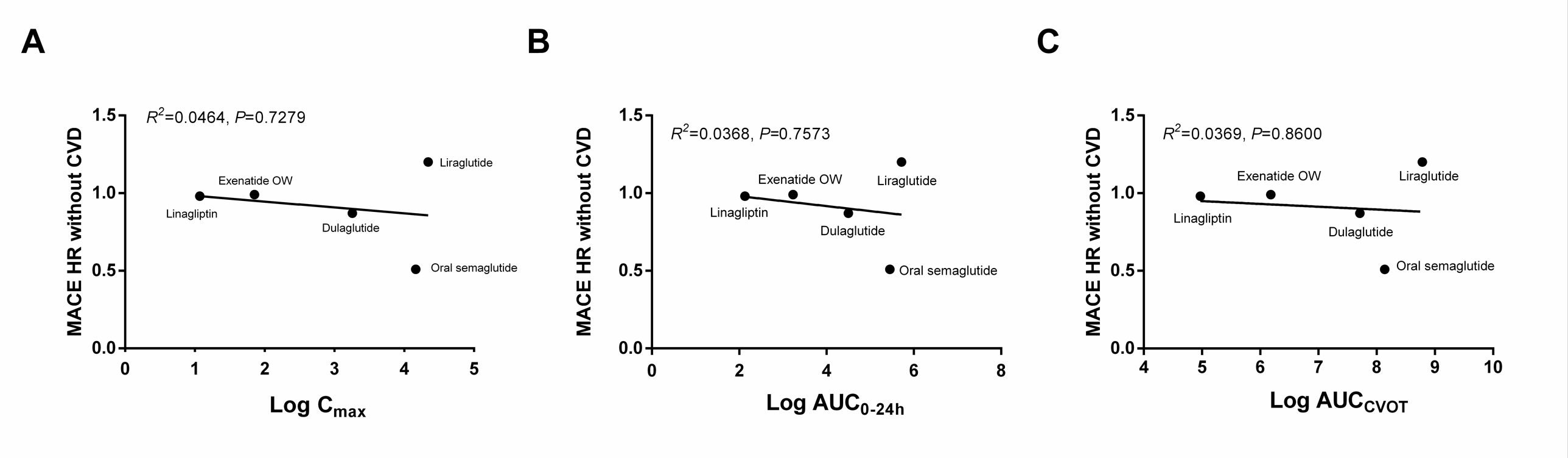


**Figure S3. Correlation between drug exposure and MACE HR without CVD.** (A) Linear regression analysis between log C_max_ and MACE HR without CVD; (B) Linear regression analysis between Log AUC_0–24h_ and MACE HR without CVD; (C) Linear regression analysis between log AUC_CVOT_ and MACE HR without CVD.





**Figure S4. Correlation between drug exposure and HbA1c reduction.** (A) Linear regression analysis between log C_max_ and HbA1c reduction; (B) Linear regression analysis between log AUC_0–24h_ and HbA1c reduction; (C) Linear regression analysis between log AUC_CVOT_ and HbA1c reduction; (D) Nonlinear regression analysis between log C_max_ and HbA1c reduction; (E) Nonlinear regression analysis between log AUC_0–24h_ and HbA1c reduction; (F) Nonlinear regression analysis between log AUC_CVOT_ and HbA1c reduction.





**Figure S5. Correlation between drug exposure and weight loss.** (A) Linear regression analysis between log C_max_ and weight loss; (B) Linear regression analysis between log AUC_0–24h_ and weight loss; (C) Linear regression analysis between log AUC_CVOT_ and weight loss; (D) Nonlinear regression analysis between log C_max_ and weight loss; (E) Nonlinear regression analysis between log AUC_0–24h_ and weight loss; (F) Nonlinear regression analysis between log AUC_CVOT_ and weight loss.





**Figure S6. Correlation between calibrated drug exposure and MACE HR.** (A) Linear regression analysis between log calibrated C_max_ and MACE HR; (B) Linear regression analysis between log calibrated AUC_0–24h_ and MACE HR; (C) Linear regression analysis between log calibrated AUC_CVOT_ and MACE HR; (D) Nonlinear regression analysis between log calibrated C_max_ and MACE HR; (E) Nonlinear regression analysis between log calibrated AUC_0–24h_ and MACE HR; (F) Nonlinear regression analysis between log calibrated AUC_CVOT_ and MACE HR. Raw data were obtained from a visual graph of the published paper. Albiglutide was excluded because no data for normalized cAMP production was available in the published paper.


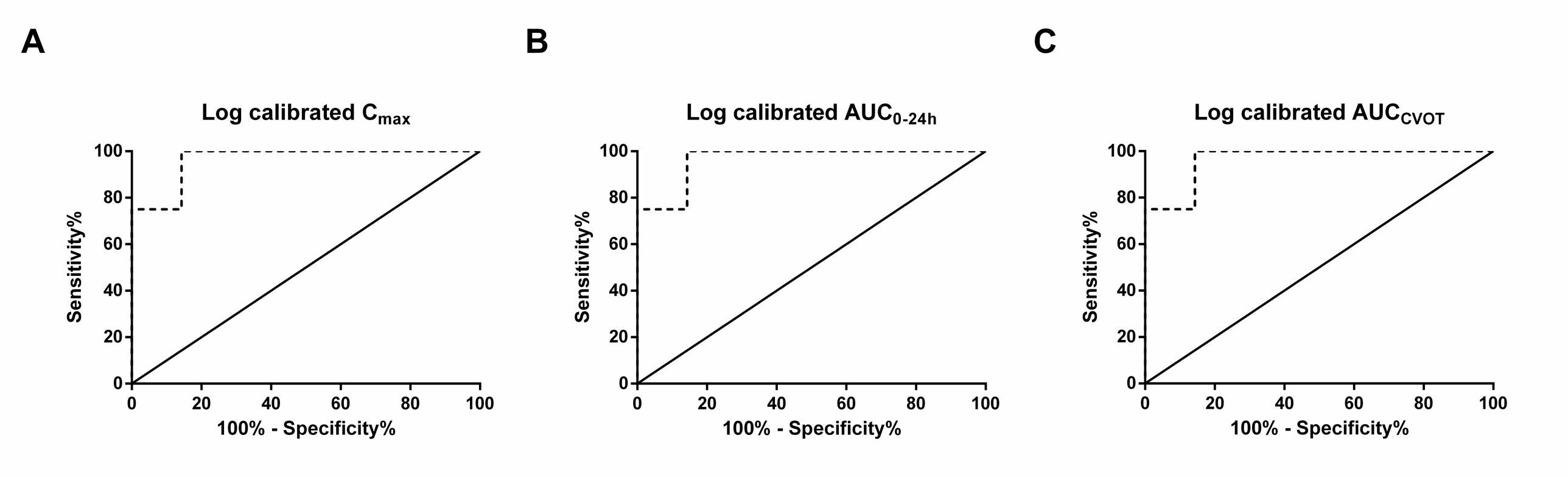


**Figure S7. Receiver operating characteristic (ROC) curves.** (A) log calibrated C_max_; (B) log calibrated AUC_0–24h_; (C) log calibrated AUC_CVOT_.





**Figure S8. Correlation between calibrated drug exposure and MACE HR with CVD.** (A) Linear regression analysis between log calibrated C_max_ and MACE HR with CVD; (B) Linear regression analysis between log calibrated AUC_0–24h_ and MACE HR with CVD; (C) Linear regression analysis between log calibrated AUC_CVOT_ and MACE HR with CVD; (D) Nonlinear regression analysis between log calibrated C_max_ and MACE HR with CVD; (E) Nonlinear regression analysis between log calibrated AUC_0-24h_ and MACE HR with CVD; (F) Nonlinear regression analysis between log calibrated AUC_CVOT_ and MACE HR with CVD.





**Figure S9. Correlation between calibrated drug exposure and HbA1c reduction.** (A) Linear regression analysis between log calibrated C_max_ and HbA1c reduction; (B) Linear regression analysis between log calibrated AUC_0–24h_ and HbA1c reduction; (C) Linear regression analysis between log calibrated AUC_CVOT_ and HbA1c reduction; (D) Nonlinear regression analysis between log calibrated C_max_ and HbA1c reduction; (E) Nonlinear regression analysis between log calibrated AUC_0–24h_ and HbA1c reduction; (F) Nonlinear regression analysis between log calibrated AUC_CVOT_ and HbA1c reduction.**

**

**Figure S10. Correlation between calibrated drug exposure and weight loss.** (A) Linear regression analysis between log calibrated C_max_ and weight loss; (B) Linear regression analysis between log calibrated AUC_0–24h_ and weight loss; (C) Linear regression analysis between log calibrated AUC_CVOT_ and weight loss; (D) Nonlinear regression analysis between log calibrated C_max_ and weight loss; (E) Nonlinear regression analysis between log calibrated AUC_0–24h_ and weight loss; (F) Nonlinear regression analysis between log calibrated AUC_CVOT_ and weight loss.

Table S1. ROC Curves of all calibrated Continuous variables

| Variable | Cutoff | P Value | Areas under the curve | Sensitivity | Specificity |
| --- | --- | --- | --- | --- | --- |
| Log calibrated C_max_ | 2.432 | 0.014 | 0.964 | 100.0 | 85.7 |
| Log calibrated AUC_0–24h_ | 3.745 | 0.014 | 0.964 | 100.0 | 85.7 |
| Log calibrated AUC_CVOT_ | 6.823 | 0.014 | 0.964 | 100.0 | 85.7 |
